# Structure and Mechanical Properties of iPP-Based Nanocomposites Crystallized under High Pressure

**DOI:** 10.3390/nano14070629

**Published:** 2024-04-04

**Authors:** Sivanjineyulu Veluri, Przemyslaw Sowinski, Mariia Svyntkivska, Zbigniew Bartczak, Tomasz Makowski, Ewa Piorkowska

**Affiliations:** Centre of Molecular and Macromolecular Studies, Polish Academy of Sciences, Sienkiewicza 112, 90 363 Lodz, Poland; siva.veluri@cbmm.lodz.pl (S.V.); przemyslaw.sowinski@cbmm.lodz.pl (P.S.); mariia.svyntkivska@cbmm.lodz.pl (M.S.); zbigniew.bartczak@cbmm.lodz.pl (Z.B.); tomasz.makowski@cbmm.lodz.pl (T.M.)

**Keywords:** isotactic polypropylene, carbon nanotubes, high-pressure crystallization, mechanical properties, plane-strain compression

## Abstract

The unique nonparallel chain arrangement in the orthorhombic γ-form lamellae of isotactic polypropylene (iPP) results in the enhancement of the mechanical properties of γ-iPP. Our study aimed at the investigation of the mechanical properties of γ-iPP nanocomposites with 1–5 wt.% multiwall carbon nanotubes (MWCNT) and 5 wt.% organo-modified montmorillonite prepared by melt-mixing and high-pressure crystallization. Neat iPP and the nanocomposites were crystallized under high pressures of 200 MPa and 300 MPa, and for comparison under 1.4 MPa, in a custom-built high-pressure cell. The structure of the materials was studied using WAXS, SAXS, DSC, and SEM, whereas their mechanical properties were tested in plane-strain compression. Under a small pressure of 1.4 MPa, polymer matrix in all materials crystallized predominantly in the α-form, the most common monoclinic form of iPP, whereas under high pressure it crystallized in the γ-form. This caused a significant increase in the elastic modulus, yield stress, and stress at break. Moreover, due to the presence of MWCNT, these parameters of the nanocomposites exceeded those of the neat polymer. As a result, a 60–70% increase in the elastic modulus, yield stress, and stress at break was achieved by filling of iPP with MWCNT and high-pressure crystallization.

## 1. Introduction

One of the most important commodity thermoplastics, isotactic polypropylene (iPP), processed under atmospheric pressure crystallizes in the most common monoclinic α-form. Specific crystallization conditions, including zone solidification [1], or the use of special nucleating agents [2,3] result in crystallization in the trigonal β-form. In turn, elevated pressure facilitates the solidification of iPP in the orthorhombic γ-form [4,5]. The γ-phase was also found in copolymers of propylene with 1-olefine-co-units [6,7,8,9], with stereo- and regio-defects [10,11,12], and in iPP of low molar mass [13,14,15]. At very high undercooling, the formation of smectic mesophase was observed [16,17]. Moreover, the trigonal δ-modification was found in the copolymers of propylene containing more than 10% of hexene or pentene comonomers [18,19], whereas in stereo-defective iPP, the orthorhombic ε-form was discovered [20].

The γ-form has received considerable attention because of its unusual structure without the parallel arrangement of chain axes. The γ-lamellae are composed of successive bi-layers, in which the parallel chain axes are inclined by approx. 80° to those in the neighboring bi-layers [21,22,23]. Interestingly, the γ-iPP crystallized under high pressure can exhibit mechanical properties different to those of α-iPP due to the unique structure of γ-crystals [24,25,26]. The Young modulus and yield stress of γ-iPP deformed in compression exceeded those of α-iPP tested in the same way [24]. The high yield stress of γ-iPP was also reported in [25,27,28]. During plane-strain compression, the main active deformation mechanisms found in the α-iPP were the crystallographic slips along the chain direction: (010)[001], (110)[001], and (100)[001] slip systems, supported by the deformation of the amorphous phase through the interlamellar shear [29]. The intense chain slip and slip instabilities resulted in the fragmentation of the lamellae into smaller crystalline blocks. Further slips in these fragmented blocks led to the strong orientation of the macromolecules along the flow direction. In turn, in the γ-iPP crystallized under high pressure, no activity of any crystallographic deformation mechanisms within the crystalline phase was detected during compression [24]. It was established that both a crystalline texture and lamella orientation developed due to the activity of the same deformation mechanism, which was the interlamellar slip due to the interlamellar amorphous shear. Numerous fine shear bands, initiated by the interlamellar shear of the amorphous layers, were observed already at the yield point [24]. The lack of crystallographic slips was suggestive of the relatively high plastic resistance of that crystallographic slip mechanism in the γ-iPP.

It is worth mentioning that not only high pressure but also high temperature is necessary to crystallize iPP in the γ-form. However, the crystallization temperature of iPP during cooling increases with the increasing pressure. Recently, a shift factor of 0.23–0.26 °C/MPa at cooling rates of 0.1–7 °C/min was determined [30,31]. Mezghani and Phillips [32] elaborated a temperature–pressure phase diagram for the α- and γ-forms of iPP, and also determined the increase in the equilibrium melting temperature (T_m_^0^) of both forms with the increasing pressure as well as the pressure dependence of the transition temperature between the α- and γ-domains. As the latter dependence is much weaker, the γ-domain broadens and the maximum undercooling for the formation of the γ-phase increases with increasing pressure. As a consequence, in iPP cooled under elevated pressure, the γ-content in the crystalline phase increased with the increasing pressure, and under the pressure of 200–300 MPa, iPP crystallized in nearly pure or pure γ-form [33]. It was also found that nucleants that can nucleate the α-phase under atmospheric pressure nucleate efficiently the γ-phase under elevated pressure [33,34]. Under lower pressure, the use of the nucleating agent shifted the crystallization temperature range to a higher temperature and therefore increased the γ-content in the crystalline phase. The predominant mechanism was the nucleation of α-lamellae, which subsequently served as seeds for the γ-lamellae via γ on α epitaxy involving the crystallographic (010)_α_ plane and the equivalent (001)_γ_ plane [34]. 

In addition, it was found that multiwall carbon nanotubes (MWCNT) nucleated the γ-phase of iPP under high pressure, whereas exfoliated organo-modified montmorillonite (o-MMT) did not exhibit such nucleating activity [35]. In our previous study [35], the addition of 1–5 wt.% of MWCNT to iPP resulted in an increase in the crystallization peak temperature during cooling by 8–13 °C, and the formation of a fine grain structure due to the MWCNT nucleation activity under elevated pressure. In turn, the iPP nanocomposites with o-MMT crystallized similarly to neat iPP. 

Modification with fillers is a widely known method of tailoring the properties of polymer materials. Among the known nanofillers, carbon nanotubes are well-recognized particles used to modify iPP properties, including thermal and mechanical properties [36]. The presence of MWCNT in the iPP matrix resulted in an increased modulus of elasticity and tensile strength due to the reinforcement effect of the nanofiller by restricting the movement of polymer chains and bearing the force themselves [37,38,39,40]. However, the studies were limited to α-iPP, whereas the influence of MWCNT on the mechanical properties of γ-iPP was not analyzed. It should be emphasized that during injection molding, iPP is subjected to elevated pressure, which facilitates the formation of the γ-phase.

This study focused on the influence of MWCNT on the mechanical properties of γ-iPP. The nanocomposites with 1–5 wt.% of MWCNT were prepared and crystallized during cooling under 200 MPa and 300 MPa. Neat iPP and iPP with 5 wt.% of o-MMT were also crystallized under the same conditions. Moreover, the isothermal crystallization of iPP and its nanocomposite with 5 wt.% of MWCNT under 200 MPa and 300 MPa was carried out. The structure and mechanical properties of these materials were examined. The mechanical properties were tested in a plane-strain compression at room temperature. The addition of 5 wt.% of MWCNT resulted in a significant increase in the elastic modulus and the yield stress of the nanocomposite in comparison with those of neat iPP. The combination of the high-pressure crystallization in the γ-form and the presence of the nanofiller resulted in properties superior to those of neat iPP crystallized in the α-phase.

## 2. Materials and Methods

### 2.1. Materials 

Isotactic polypropylene (iPP) Adstif HA740N (melt flow rate of 12 g/10 min at 230 °C/2.16 kg, density of 0.9 g/cm^3^) was purchased from Basell Orlen Polyolefins (Płock, Poland). Anox 20 and Ultranox 626, used to stabilize iPP, were supplied by Addivant (Danbury, CT, USA). Multiwall carbon nanotubes (MWCNT), and organo-modified montmorillonite (o-MMT) for comparison, were used as nanofillers. iPP masterbatch with 20 wt.% of MWCNT Plasticyl PP2001 with a density of 0.872 g/cm^3^ was purchased from Nanocyl (Sambreville, Belgium). According to the supplier, the average diameter of MWCNT NC7000 was 9.5 nm, the average length 1.5 µm, carbon purity 90%, and surface area 250–300 m^2^/g [41]. For comparison, o-MMT Cloisite C15A purchased from Southern Clay Products (Gonzales, TX, USA) was also used. According to the supplier, it was modified with dimethyl di(hydrogenated tallow) quaternary ammonium. Its particle size was below 13 µm, whereas its d-spacing was 3.15 nm. To compatibilize o-MMT with iPP, maleic anhydride-grafted polypropylene (PP-g-MA), Polybond 3150 from Chemtura Corporation (Shelton, CT, USA), with 0.5 wt.% of MA groups, density of 0.91 g/cm^3^, and MFR of 50 g/10 min (230 °C/2.16 kg) was used.

### 2.2. Nanocomposite Preparation 

Nanocomposites of iPP with 1, 3, 5 wt.% of MWCNT and 5 wt.% of o-MMT denoted as PP/CN1, PP/CN3, PP/CN5, and PP/MT5, respectively, were prepared by melt mixing in a Brabender (Duisburg, Germany) batch mixer. To increase its stability against degradation, iPP was mixed with 0.2 wt.% of Anox 20, 0.1 wt.% of Ultranox 626, and 0.2 wt.% of calcium stearate, at 195 °C, for 6 min at 60 rpm. To obtain PP/CN nanocomposites with a desirable content of nanofillers, iPP was mixed with appropriate amounts of Plasticyl PP2001 masterbatch. To obtain PP/MT5, at first masterbatch of o-MMT with PP-g-MA (1/2 *w*/*w*) was prepared as it was described elsewhere [35], and then it was added to iPP. While compounding of iPP with the masterbatches, the rotation speed was increased within 4 min, every minute by 10 rpm, and the mixing was continued for the next 10 min at 100 rpm. Neat iPP was processed in the same way to obtain a reference material. The detailed description of the nanocomposite preparation and structure, characterized by transmission electron microscopy and X-ray diffraction, was described by us previously [35]. A good dispersion of MWCNT and o-MMT was achieved in PP/CN and PP/MT5 nanocomposites, respectively, and in the latter o-MMT, platelets were exfoliated.

### 2.3. Crystallization 

**Figure 1:**
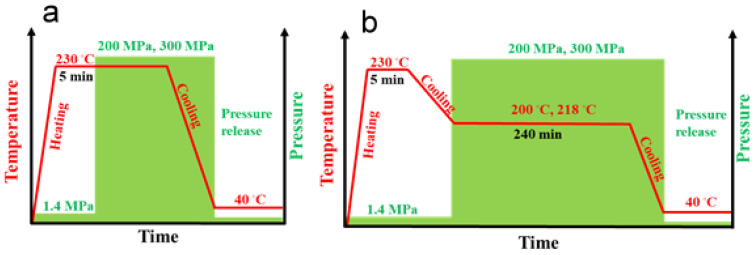
Schemes of pressure and temperature protocols of nonisothermal crystallization (**a**) and isothermal crystallization (**b**).

To crystallize materials under high pressure in the γ-form, we used a special custom-built steel cell, with a barrel and pistons, heaters, and sensors, as described elsewhere [33,42]. The temperature and pressure protocols for nonisothermal and isothermal crystallization are presented in Figure 1. The specimens (compression molded 1 mm thick disks assembled in approx. 2 g cylinders with approx. 9.5 mm diameters) were placed in the cell, and to ensure good thermal contacts, a small pressure of 1.4 MPa was applied. Then, the specimens were heated under 1.4 MPa to 230 °C, and after 5 min at 230 °C, the molten polymer in the cell was pressurized to 200 MPa or 300 MPa using an Instron 5582 testing machine (Instron Corp., High Wycombe, UK) at a cross-head speed of 2 mm/min, through a fixture stabilizing the load precisely along the cell axis. Then, the cell was cooled to approx. 40–50 °C, the pressure was released, and the specimens were removed from the cell. In addition, neat iPP and PP/CN5 nanocomposite were crystallized isothermally at 200 °C under 200 MPa, and at 218 °C under 300 MPa. After 5 min at 230 °C under 1.4 MPa, the temperature was decreased to 200 °C or 218 °C, and the pressure was increased to 200 or 300 MPa, respectively. These temperatures were selected based on T_m_^0^ values measured by others [32,43] and extrapolated to 300 MPa as described in [34]. After 4 h at isothermal conditions, the specimens were cooled to approx. 40–50 °C, and the pressure was released. To obtain reference materials with an iPP crystalline phase in the α-form, all the materials were crystallized during cooling under a pressure of 1.4 MPa. The constant hydrostatic pressure and temperature inside the cell were controlled with an accuracy of ± 0.5 MPa and 1 °C, respectively. Of note, the cooling rate of the cell, although not controlled, was reproducible, at 5–8 °C/min in the temperature range of iPP crystallization. 

### 2.4. Characterization

The crystallized samples were analyzed by wide-angle and small-angle X-ray scattering (WAXS and SAXS) and differential scanning calorimetry (DSC), including fast scanning DSC (FS-DSC), and scanning electron microscopy (SEM).

The crystallographic structure and crystallinity degree (*X_c_*) in the crystallized specimens were examined by WAXS in a reflection mode using an Aeris diffractometer (Malvern Panalytical Ltd., Malvern, UK), operating at 40 kV and 7.5 mA, with CuKα radiation (0.154056 nm). The diffractograms were recorded in a 2θ range of 10–70° with a step of 0.022° and then deconvoluted using the WAXSFIT 4.0 program (ATH, Bielsko-Biala, Poland) [44], as described by us elsewhere [33]. The content of the α and γ-forms, K_α_ and K_γ_, in the crystalline phase was determined by taking advantage of the equations proposed by Turner-Jones et al. [45], based on the integral intensities (I) of reflections from the crystallographic planes (117)_γ_ and (130)_α_:

(1) $$K_{\gamma }={I(117)}_{\gamma }\;{\left[{I(117)}_{\gamma }+{I(130)}_{\alpha }\right]}^{-1}$$

(2) $$K_{\alpha }=1-K_{\gamma }$$

The amorphous halos were also obtained by deconvolution, and values of X*_c_* were evaluated. 

The lamellar structure of the crystallized materials was probed using 2-dimensional SAXS (2D-SAXS). A 1.2 m long Kiessig-type SAXS camera was coupled to an X-ray CuKα low divergence micro-source from GeniX Cu-LD Xenocs (Grenoble, France), operating at 50 kV and 1 mA. The patterns were recorded with a Pilatus 100K solid-state detector (Dectris, Switzerland). The average long period (L_p_) values were deduced from the positions of peaks in Kratky plots, according to the Bragg law. The average lamella thicknesses (L_cx_) were calculated based on L_p_ and X_c_, the latter recalculated to volume crystallinity assuming the densities of the amorphous phase (d_a_) of 0.855 g/cm^3^ and the crystalline α and γ-phases (d_cα_ and d_cγ_) of 0.936 g/cm^3^ and 0.938 g/cm^3^ [46], respectively.

The melting behavior of the crystallized materials was analyzed with DSC Q20 from TA Instruments (New Castle, DE, USA). Approx. 4 mg specimens of all materials were heated at 10 °C/min from 25 °C to 230 °C. In addition, a FS-DSC Mettler Toledo Flash DSC 2+ (Greifensee, Switzerland) was used to perform fast scanning calorimetry measurements. The sensors employed (Mettler Toledo Multistar UFS1) were at first conditioned and temperature-verified according to the instrument specification. Specimens, of approx. 150 ng, were prepared by cutting thin sections from crystallized materials using a microtome. The obtained specimens were placed in the centers of the sensors. Heating to 230 °C was conducted at a heating rate of 5000 °C/min. The measurements were carried out in a nitrogen atmosphere. 

The heating thermograms were used to evaluate the melting enthalpy by the integration of the melting peaks, and also to determine the lamella thickness of the materials. The Gibbs–Thomson equation [47] expresses the dependence of melting temperature (T) of plate-shaped lamella on its thickness (L):

(3) $$T=T_{m}^{0}\left[1-2\sigma_{e}\;{\left(\Delta H_{c}\;d_{c}\;L\right)}^{-1}\right]$$

where T_m_^0^ is the equilibrium melting temperature, σ_e_ is the surface free energy of the lamella basal plane, ΔH_c_ is the heat of fusion of crystals per unit mass, and d_c_ is the crystal density. In turn, Crist and Mirabella [48] proposed a method for the determination of an average lamella thickness (L_av_) based on DSC thermograms, which was successfully used by others [25,49]. According to [48], the weight fraction of crystals with thicknesses between L and L + dL, denoted as g(L)dL, which melts between T and T + dT, can be expressed by the following formula:

(4) $$g\left(L\right)dL=P\left(T\right)\;dT\;{\left[X_{c}\;\Delta H_{c}\;M\;(dT/dt)\right]}^{-1}$$

where: X_c_ is the weight crystallinity, M is the sample weight, P(T) is the power absorbed at temperature T, and t is time. Evaluating dT/dL based on Equation (3), and substituting in Equation (4) yields the following:

(5) $$g\left(L\right)=A\;P\left(T\right)\;{\left(T_{m}^{0}-T\right)}^{2}$$

where: T=T(L) is as described by Equation (3), and A is a normalizing constant equal to $d_{c}{\left[2\sigma_{e}T_{m}^{0}MX_{c}(dT/dt)\right]}^{-1}$. L_av_ is expressed by the integral over the entire range of L, (L_min_; L_max_):

(6) $$L_{av}=\int_{L_{min}}^{L_{max}}L\;g\left(L\right)\;dL$$

Equations (3), (5) and (6) were used to determine L_av_ based on the DSC melting exotherms. In the calculations, T_m_^0^, σ_e_, and ΔH_c_ of the α- and γ-form equal to 459.25 K (186.1 °C), 209 J/g, 0.0522 J/m^2^, and 460.35 K (187.2 °C), 190 J/g, and 0.0517 J/m^2^ [32], respectively, were used, and the d_c_ values were as mentioned previously. These values were selected as resulting in lamella thicknesses closest to the L_cx_ obtained by X-ray methods.

Plane-strain compression tests were performed using an Instron 5582 testing machine (High Wycombe, UK) and a compression tool of channel-die type, equipped with a strain gauge. The tool consisted of a lower die with a wide rectangular channel and an upper plunger fitting the channel inside the lower die, as described in detail elsewhere [24,50]. The sizes of the die channel were the following: width of 8.1 mm (along the constrained direction, CD), length of 3.2 mm (along the flow direction, FD), and depth of 4 mm (along the loading direction, LD), which allowed specimens up to 4 mm high to be compressed. The specimens for the compression experiments were cut from the crystallized samples by precise machining to the form of cuboids 9 mm long, 8.1 mm wide, and 4 mm high, and then stored at room temperature for 3 days. To mitigate friction during testing, a lubricant was applied on the specimen surfaces contacting the die and the plunger. The plane-strain compression experiments were performed at a constant true strain rate of 0.05/min at room conditions. To determine the average mechanical parameters, the tests were repeated at least five times for each type of sample. 

To gain an insight into the internal structure of undeformed and deformed specimens, they were cut with an ultramicrotome, and exposed surfaces were permanganate etched according to the method developed by Olley et al. [51] and used also by others [24,34]. The etchant contained 0.7 *v*/*v* of KMnO_4_, dissolved in a 5:4:1 *v*/*v*/*v* mixture of 95% sulfuric acid, 85% phosphoric acid, and distilled water. To improve the etching, the specimens submerged in the etching liquid were subjected to periodic sonication for short times. After 1.5–2 h etching at room temperature, washing, and drying, the specimens were sputtered with gold and studied with SEM JEOL 6010LA (Tokyo, Japan), operating in a high-vacuum mode at an accelerating voltage of 10 kV. iPP and PP/CN5 specimens nonisothermally crystallized under 200 MPa and 300 MPa were analyzed before deformation and also after compression to a true strain of 0.2, 0.4, and 0.6. 

## 3. Results and Discussion

### 3.1. Structure

**Figure 2:**
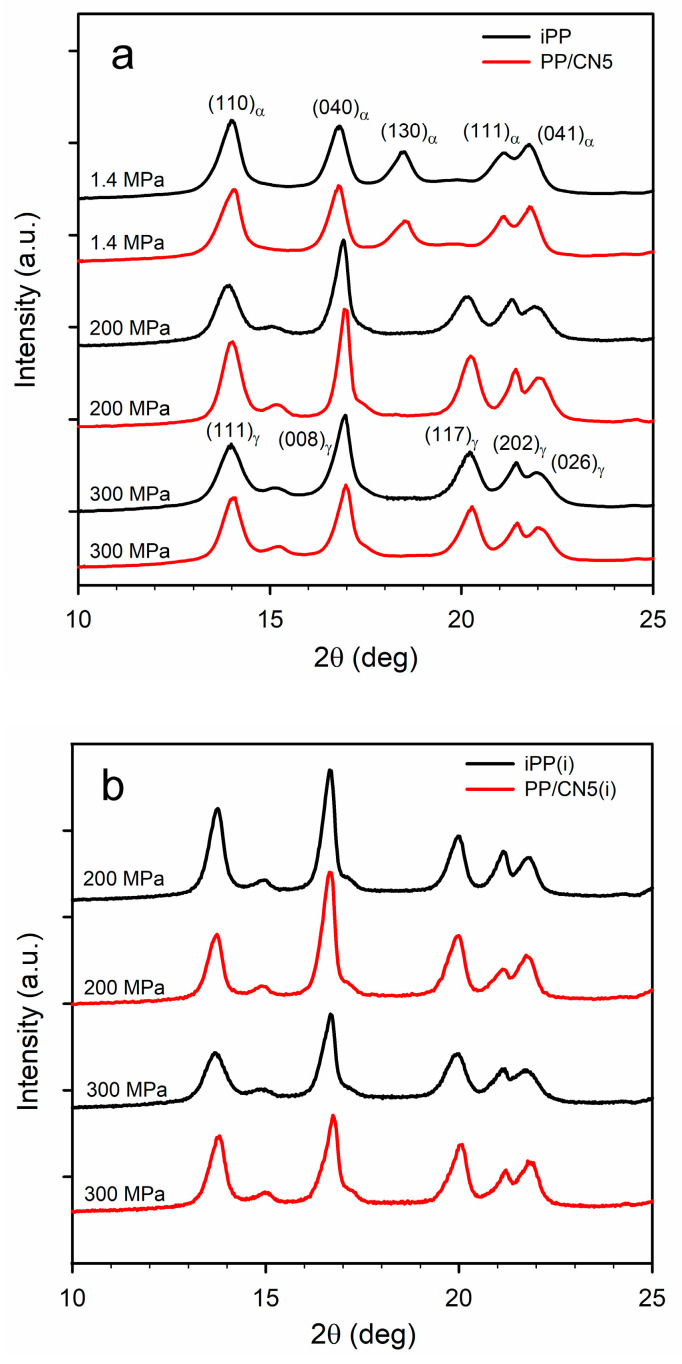
(**a**) WAXS curves of iPP and PP/CN5 crystallized nonisothermally under 1.4 MPa, 200 MPa, and 300 MPa. (**b**) WAXS curves of iPP and PP/CN5 crystallized isothermally, as denoted by (i), under 200 MPa and 300 MPa.

Exemplary WAXS curves of the materials are shown in Figure 2a,b. WAXS curve of each material nonisothermally crystallized under 1.4 MPa exhibited a pronounced (130)_α_ peak and only either a weak or trace (117)_γ_ peak, as these materials contained the predominant α-phase. K_γ_ calculated based on Equation (1) was only of 0.10–0.16, being the highest for PP/CN3 and PP/CN5. On the contrary, WAXS curves of iPP and PP/CN crystallized nonisothermally under 200 MPa and 300 MPa exhibited (117)_γ_ peak, whereas (130)_α_ peak was absent, and K_γ_ was equal to 1.0. The same applies to PP/MT5 crystallized under 300 MPa. Only for PP/MT5 crystallized under 200 MPa, a very weak (130)_α_ peak was observed, which resulted in K_γ_ of 0.96. The X_c_ of nonisothermally crystallized materials was similar, regardless of the pressure, as is shown in Table S1 in the Supplementary Materials. It was in the range of 57–60% for iPP and 53–57% for PP/MT5. PP/CN crystallized under 1.4 MPa exhibited an X_c_ of 59–61%, whereas X_c_ of those crystallized under 200 MPa and 300 MPa reached 61–65%. These results are similar to those previously reported [35]. In the WAXS curves of iPP and PP/CN5 crystallized isothermally under 200 MPa and 300 MPa, the (117)_γ_ peak was visible, whereas the (130)_α_ peak was absent, and K_γ_ was equal to 1. Crystallization under these conditions resulted in a higher X_c_ of 69–71%.

**Figure 3:**
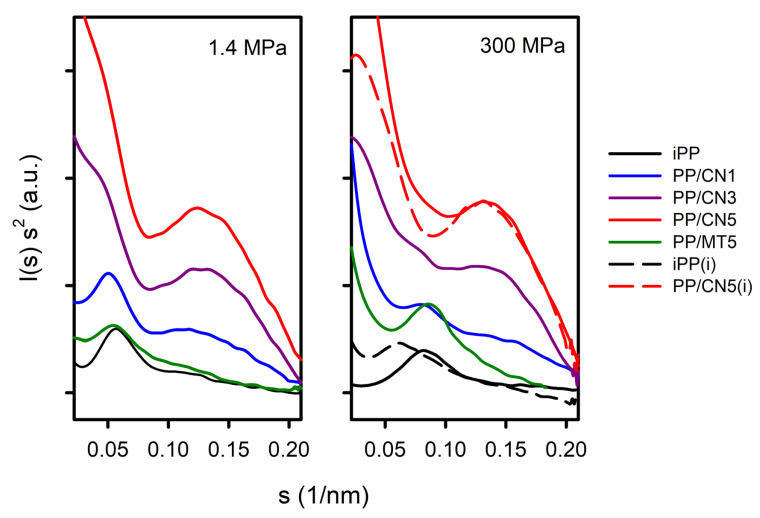
Kratky plots of I(s) s^2^ vs. s. I(s) is SAXS intensity and s = 2 sin (θ)/λ, where λ is wavelength. (i) denotes materials crystallized isothermally.

Figure S1 in Supplementary Materials shows 2D-SAXS patterns. Exemplary Kratky plots are shown in Figure 3. The pronounced maxima, attributed to the scattering from the periodicity of the polymer structure, were visible only in the Kratky plots of iPP, PP/MT5, and PP/CN1. L_p_ and L_cx_ values, the latter calculated based on L_p_ and X_c_, are listed in Table S1 in Supplementary Materials. L_p_ and L_cx_ of the materials crystallized under 1.4 MPa in the predominant α-form were in the range of 18.2–19.4 nm and 10–11.4 nm, respectively. Smaller values of L_p_ and L_cx_ were obtained for materials crystallized nonisothermally under high pressure in the γ-form, 11.5–13.9 nm, and 5.9–8.2 nm, respectively. The lowest values were obtained for PP/MT5, which may result from about 10 wt.% of PP-g-MA content in the nanocomposite. In turn, L_p_ and L_cx_ values of iPP crystallized isothermally under 200 MPa were 16.9 nm and 11.5 nm, respectively, whereas those of iPP crystallized under 300 MPa were 16.0 nm and 10.7 nm. With the increasing MWCNT content, the scattering from the nanofiller became stronger and obscured that from the periodicity of the polymer. However, additional maxima appeared on the Kratky plots of PP/CN nanocomposites, suggestive of scattering objects with sizes of 7.5–8.5 nm, which are close to the average MWCNT diameter of 9.5 nm.

**Figure 4:**
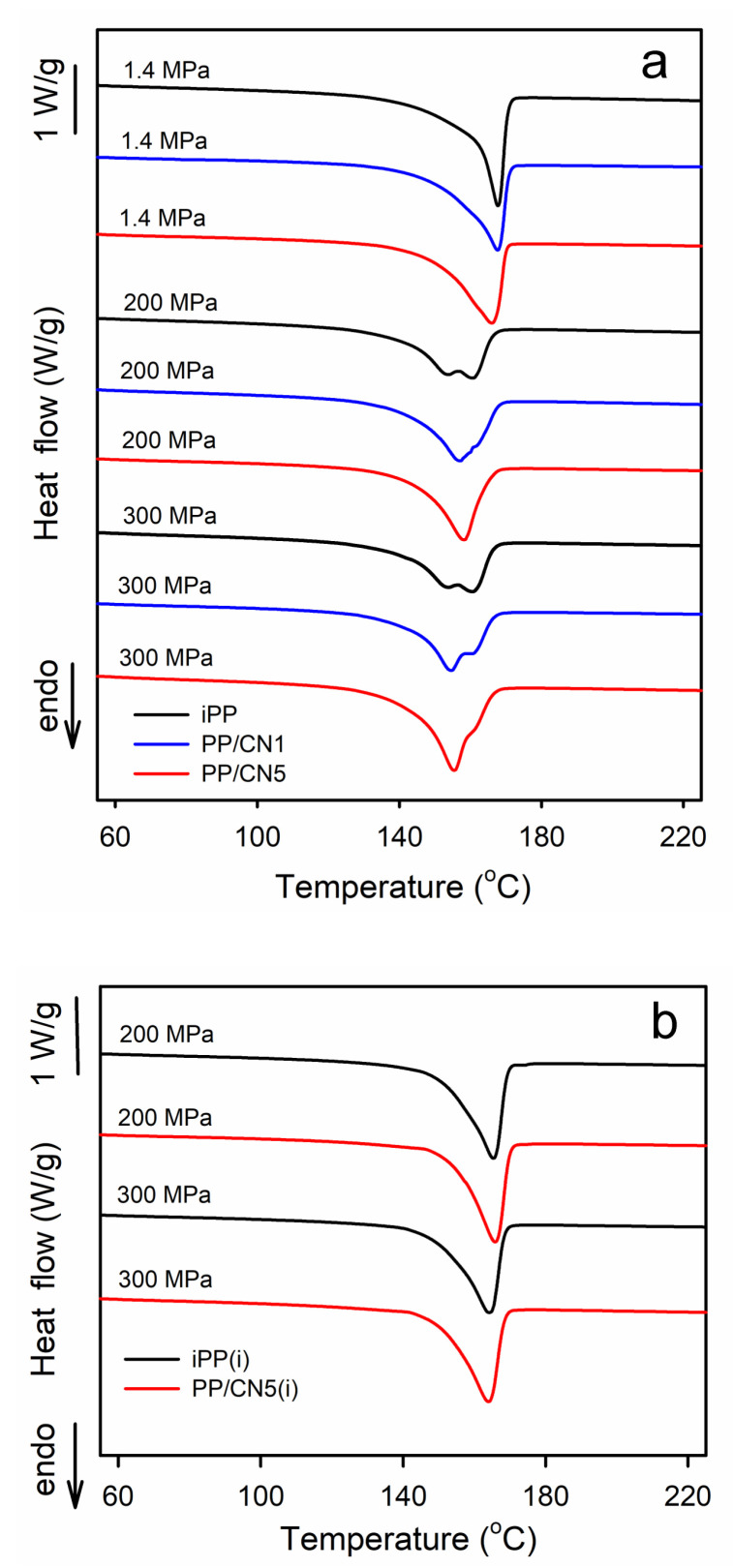
(**a**) DSC heating thermograms of iPP, PP/CN1, and PP/CN5 crystallized nonisothermally under 1.4 MPa, 200 MPa, and 300 MPa. (**b**) DSC heating thermograms of iPP and PP/CN5 crystallized isothermally, as denoted by (i), under 200 MPa and 300 MPa. Heating rate of 10 °C/min.

Exemplary DSC heating thermograms recorded at 10 °C/min, collected in Figure 4a, show the evolution from the melting of the predominant α-form to the melting of the pure γ-form. The melting endotherms of all materials crystallized under 1.4 MPa were featured with single melting peaks with T_m_ of 165–168 °C. iPP and PP/MT5 crystallized during cooling under 200 and 300 MPa exhibited double-peak melting behavior, with T_m_ at about 160 °C and 155 °C. The T_m_ of nonisothermally crystallized PP/CN also decreased with the increasing crystallization pressure, to about 158 °C at 200 MPa and to 155 °C at 300 MPa. Moreover, the melting peaks of these nanocomposites exhibited shoulders on their descending slopes. The melting enthalpy, ΔH_m_, also decreased with the increasing crystallization pressure, from 101 to 107 J/g_PP_ of the materials crystallized under at 1.4 MPa to 89 to 94 J/g_PP_ for the same materials cooled under 200 and 300 MPa, as ΔH_c_ of the α-form exceeds that of the γ-form. Such complex melting behavior was reported by us previously [35] and interpreted as being related to the reorganization phenomena in the materials during heating in DSC experiments. In turn, iPP and PP/CN5 isothermally crystallized in the γ-form under 200 and 300 MPa exhibited single melting peaks, shown in Figure 4b, with T_m_ at 164–166 °C and ΔH_m_ of 100–105 g/J_PP_, which supports their higher X_c_ determined by WAXS. 

The average lamella thicknesses, L_av_, determined based on the DSC thermograms and Equations (3), (5) and (6), are listed in Table S1 in the Supplementary Materials. It appears that the L_av_ values agreed with the L_cx_ determined by the X-ray method. The L_av_ of iPP and the nanocomposites crystallized under 1.4 MPa was 9.3 – 10.3 nm, whereas 7.9 – 8.4 nm of these materials cooled under 200 and 300 MPa, confirming the smaller thickness of the γ-lamellae. In turn, the L_av_ values of iPP and PP/CN5 lamellae crystallized isothermally, 9.7 – 10.6 nm, exceeded those of crystals formed in these materials during cooling under the same pressure of 200 and 300 MPa.

Selected iPP and PP/CN5 were studied by FS-DSC at 5000 °C/min to estimate the possible influence of the reorganization in the crystalline phase during heating on the obtained L_av_ values. Exemplary FS-DSC thermograms are shown in Figure S2 in Supplementary Materials. All thermograms exhibited single melting peaks. During fast heating, the T_m_ of iPP decreased by 8 °C at 1.4 MPa. In the case of iPP crystallized under 200 and 300 MPa, T_m_ was 5–7 °C below the T_m_ of its high-temperature melting peak, recorded at 10 °C/min, at 161 °C. The T_m_ decrease was smaller for PP/CN5, below 3 °C. However, L_av_ values determined based on the FS-DSC thermograms were similar to those based on the melting exotherms recorded at 10 °C/min and confirmed that the γ-lamellae in the materials nonisothermally crystallized under high pressure were thinner than the α-lamellae formed in the same materials during cooling under 1.4 MPa. 

**Figure 5:**
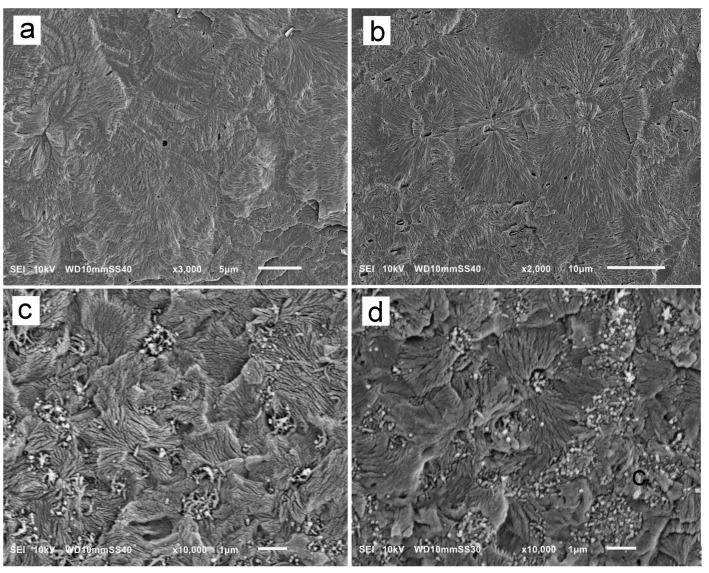
SEM micrographs of iPP nonisothermally crystallized under 200 MPa (**a**) and 300 MPa (**b**), and PP/CN5 nonisothermally crystallized under 200 MPa (**c**) and 300 MPa (**d**).

Exemplary SEM micrographs of permanganate-etched iPP and PP/CN5 crystallized during cooling under 200 MPa and 300 MPa are shown in Figure 5. SEM analysis confirmed the presence of polycrystalline aggregates in the materials. In iPP crystallized under 200 MPa, the aggregates, with sizes of up to 30 µm, did not resemble typical spherulites. In addition to the fans protruding from nucleation sites, there were stacks of parallel γ-lamellae nucleated on longer α-lamellae, as shown in Figure 5a. Occasionally, the cross-hatching typical of the α-form was observed, although these α-lamellae were densely overgrown with γ-lamellae. In iPP crystallized under 300 MPa, more aggregates resembled typical spherulites, with γ-lamellae protruding from nucleation sites as shown in Figure 5b, although the lamella stacks were also observed. Such features of the semicrystalline morphology were previously observed and described [5,24,34]. As the α-lamellae served only as seeds for the γ-phase, their content was far too small to be detected by WAXS, as is in the present study.

On the SEM micrographs of PP/CN5, in Figure 5c,d, in addition to polycrystalline aggregates, MWCNT and their remnants left after etching were visible. The polycrystalline aggregates were significantly smaller than those in neat iPP due to the nucleation activity of MWCNT under high pressure [35]. Frequently, they were in the form of lamella fans, with sizes mostly up to 2 μm, protruding from nucleation sites, but seldom forming spherulite-like structures. In addition, stacks of parallel lamellae were observed, especially in PP/CN5 crystallized under 200 MPa, suggestive of the nucleation of the γ-lamellae on α-lamellae.

### 3.2. Mechanical Behavior

**Figure 6:**
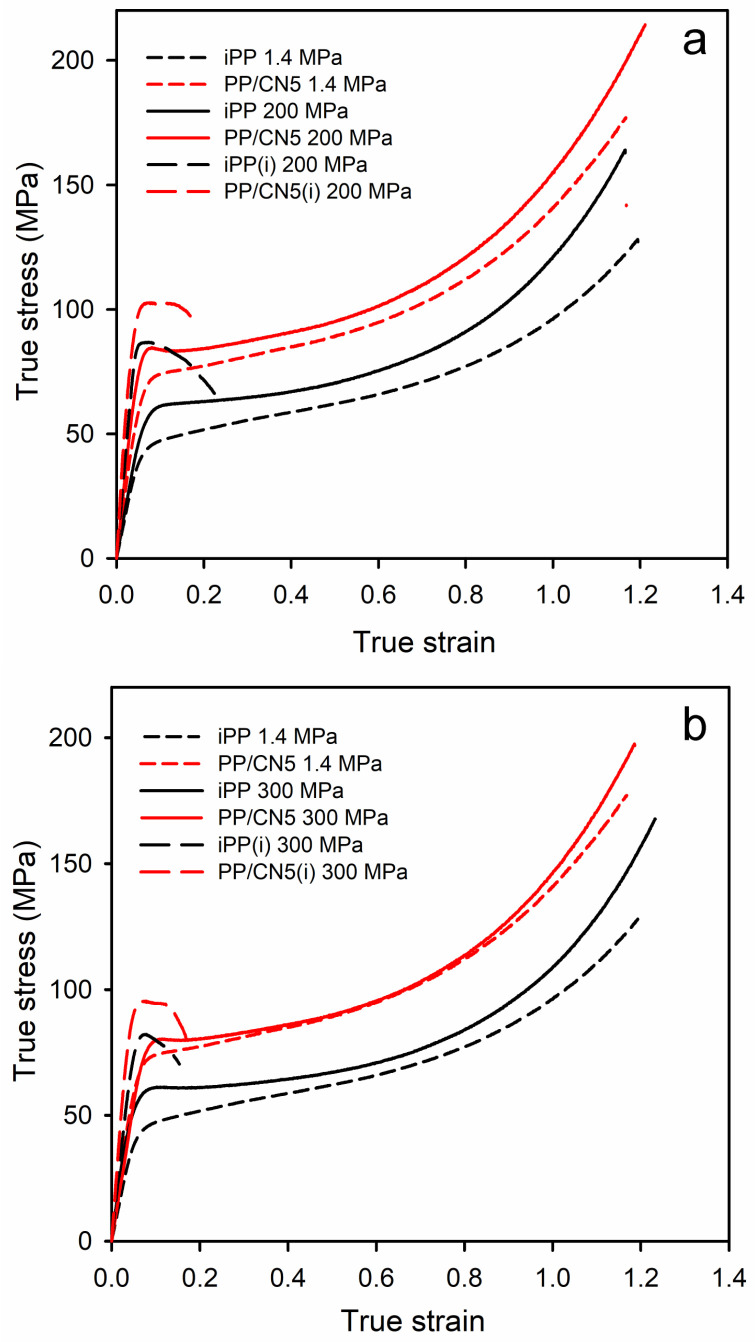
Comparison of true stress–true strain dependencies of iPP and PP/CN5: crystallized under 1.4 MPa and 200 MPa (**a**), crystallized under 1.4 MPa and 300 MPa (**b**). (i) denotes materials crystallized isothermally.

The exemplary true stress–true strain dependencies of the materials studied are shown in Figure 6, and in Figure S3 in Supplementary Materials, whereas the relevant mechanical parameters are collected in Table 1. Regardless of the crystallization pressure, PP/CN nanocomposites, especially with 3 wt.% and 5 wt.% of MWCNT, exhibited a higher elastic modulus (E), yield stress (σ_y_), and stress at break (σ_b_) compared to neat iPP crystallized under the same conditions because of restricting the movement of polymer chains and bearing the force by the nanofiller. In the case of nonisothermally crystallized materials, the strength was determined by σ_b_, as the stress increased during deformation due to the hardening. The E, σ_y_, and σ_b_ of PP/CN increased with the increasing MWCNT content. In turn, the modification of iPP with 5 wt.% of o-MMT did not result in a significant improvement in these mechanical parameters. Moreover, all the materials crystallized nonisothermally under high pressure in the γ-form exhibited significantly higher E, σ_y_, and σ_b_ than those crystallized under 1.4 MPa in the predominant α-form. In turn, the values of strain at break (ε_b_) were similar, approx. 1.2, regardless of the composition and crystallization pressure. In comparison to neat iPP crystallized under 1.4 MPa, the filling with 5 wt.% of MWCNT and crystallization during cooling under 200 MPa caused an increase in E, σ_y_, and σ_b_ from 1290 MPa, 50 MPa, and 129 MPa to 2050 MPa, 84 MPa, and 213 MPa, respectively. This is an increase of approx. 60%, 70%, and 65%, respectively. The isothermally crystallized iPP and PP/CN5 exhibited even higher E and σ_y_ than the same materials crystallized during cooling due to higher crystallinity and a somewhat larger lamella thickness. The E and σ_y_ of PP/CN5 isothermally crystallized under 200 MPa were 2550 MPa and 101 MPa, respectively, and exceeded by approx. 100% those of iPP crystallized during cooling under 1.4 MPa. However, this increase was achieved at the expense of ε_b_ and σ_b_. ε_b_ was less than 0.25, whereas σ_b_ was below σ_y_. This early fracture was most likely due to the weak bonding between amorphous and crystalline phases and between polycrystalline aggregates resulting from crystallization at high temperatures. It should be noted that crystallization during cooling under 200 MPa resulted in the higher E and σ_y_ of the materials than crystallization under 300 MPa, despite the same γ-form of the polymer crystalline phase, the similar X_c_, and lamella thickness. We hypothesize that these differences may be related to the expansion of the polymer after releasing the pressure.

**Figure 7:**
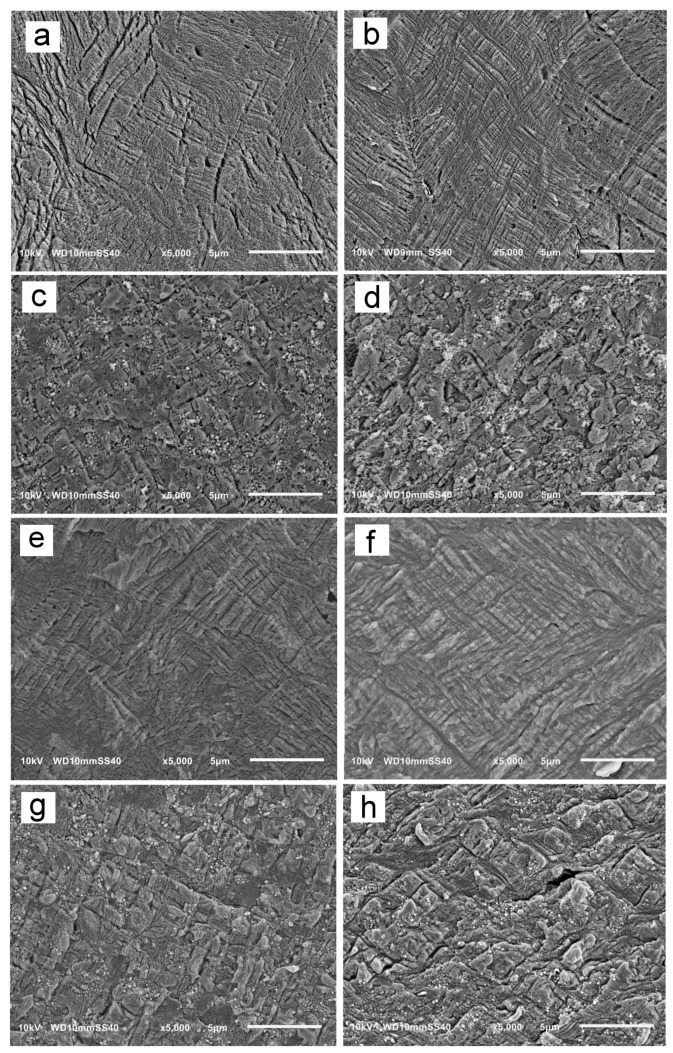
SEM micrographs of iPP and PP/CN5 crystallized nonisothermally under 200 MPa and 300 MPa, and compressed to true strain of 0.2 and 0.6: iPP, 200 MPa, 0.2 (**a**), iPP, 300 MPa, 0.2 (**b**), PP/CN5, 200 MPa, 0.2 (**c**), PP/CN5, 300 MPa, 0.2 (**d**), iPP, 200 MPa, 0.4 (**e**), iPP, 300 MPa, 0.4 (**f**), PP/CN5, 200 MPa, 0.6 (**g**), PP/CN5, 300 MPa, 0.6 (**h**). LD vertical.

Previously [24], the increase in σ_y_ of γ-iPP in comparison to α-iPP was attributed to the different mechanisms of the plastic deformation. In the γ-iPP crystallized under high pressure, no crystallographic deformation mechanisms within the crystalline phase were detected during compression. This was most probably because of the unique nonparallel chain arrangement in the orthorhombic γ-form of iPP. The interlamellar slip due to the interlamellar amorphous shear was identified as the main mechanism. Hence, to have an insight into the mechanism of plastic deformation of the γ-phase of the iPP matrix in the presence of MWCNT, specimens of PP/CN5 nonisothermally crystallized under 200 MPa and 300 MPa were compressed to a true strain of 0.2, 0.4, and 0.6, and analyzed using SEM. Neat iPP crystallized under the same conditions was also examined for comparison. Figure 7 and Figure S4 in Supplementary Materials show exemplary SEM micrographs of deformed specimens cut parallel to the LD-FD plane and permanganate etched.

In all tested materials, shear bands, inclined at 45° to LD, were visible at a strain of 0.2 and also at larger strains of 0.4 and 0.6. This shows that the presence of MWCNT did not alter the mechanism of deformation. However, the shear bands in PP/CN5 seemed to be shorter than in iPP deformed to the same strain. This may be a result of the hindrance to the propagation of shear bands due to the presence of MWCNT. This hindrance could contribute to the increase in the σ_y_ of PP/CN5 crystallized under high pressure in the γ-form.

## 4. Conclusions

Our study aimed at the investigation of the mechanical properties of γ-iPP nanocomposites. Neat iPP and nanocomposites of iPP with 1–5 wt.% of MWCNT, and for comparison with 5 wt.% of o-MMT, were prepared and crystallized in the orthorhombic γ-form. The preparation of these materials comprised the following steps: (i) compounding of the components and (ii) crystallization under high pressure of 200 MPa and 300 MPa, according to the protocols presented in Figure 1. The materials were also crystallized under 1.4 MPa, in the predominant α-form, for comparison. All materials were crystallized during cooling. In addition, iPP and PP/CN5 were crystallized isothermally at 200 °C under 200 MPa and at 218 °C under 300 MPa. Regardless of the pressure, PP/CN nanocomposites, especially PP/CN3 and PP/CN5, exhibited higher E, σ_y_, and σ_b_ than neat iPP, whereas the filling of iPP with 5 wt.% of o-MMT did not result in a significant improvement in these mechanical parameters. Moreover, neat iPP and the nanocomposites crystallized during cooling under high pressure in the γ-form exhibited significantly higher E, σ_y_, and σ_b_ than those crystallized under 1.4 MPa in the predominant α-form. Although the increase in E and σ_y_ was even higher in the case of isothermally crystallized materials, this was at the expense of ε_b_, which drastically decreased. Moreover, crystallization under 200 MPa resulted in a higher E and σ_y_ of the materials than crystallization under 300 MPa, despite the same γ-form of the polymer crystalline phase, similar X_c_, and lamella thickness. Thus, the filling of iPP with 5 wt.% of MWCNT by compounding of the components, and crystallization in the γ-form during cooling under 200 MPa, increased the E, σ_y_, and σ_b_, by approx. 60%, 70%, and 65%, respectively. Based on the analysis of the structure of deformed specimens of iPP and PP/CN5, it was concluded that the presence of MWCNT did not alter the mechanism of the plastic deformation during the plane-strain compression of the iPP matrix crystallized in the γ-phase. Nevertheless, MWCNT hindered the propagation of the shear bands, which are crucial for this process, and this may contribute to the increase in σ_y_ of PP/CN nanocomposites with the polymer matrix solidified in the γ-form.

## Figures and Tables

**Table 1:** Mechanical parameters of iPP, PP/CN, and PP/MT5 crystallized nonisothermally under 1.4 MPa, 200 MPa, and 300 MPa, iPP, and PP/CN5 crystallized isothermally under 200 MPa and 300 MPa: E—elastic modulus, σ_y_—yield stress, σ_b_ and ε_b_—stress and strain at break, respectively. σ_y_ was determined with 3% offset. (i) denotes materials crystallized isothermally.

Sample Code		1.4 MPa				200 MPa				300 MPa		
	E [MPa]	σ_y_ [MPa]	σ_b_ [MPa]	ε_b_	E [MPa]	σ_y_ [MPa]	σ_b_ [MPa]	ε_b_	E [MPa]	σ_y_ [MPa]	σ_b_ [MPa]	ε_b_
iPP	1290	50	129	1.20	1500	62	165	1.18	1430	59	185	1.23
PP/CN1	1360	67	132	1.18	1530	77	176	1.15	1440	74	176	1.17
PP/CN3	1430	72	156	1.18	1730	80	199	1.22	1590	77	188	1.19
PP/CN5	1620	74	178	1.16	2050	84	213	1.21	1830	79	185	1.18
PP/MT5	1320	52	125	1.17	1560	61	156	1.19	1370	60	174	1.22
iPP(i)	-	-	-	-	2100	86	69	0.21	1680	81	72	0.16
PP/CN5(i)	-	-	-	-	2550	101	96	0.15	2390	94	82	0.16

## Data Availability

The data presented in this study are available on request from the corresponding author.

## Supplementary Materials

The following supporting information can be downloaded at: https://www.mdpi.com/article/10.3390/nano14070629/s1, Table S1: Characteristics of iPP and its nanocomposites, PP/CN and PP/MT5, crystallized under pressure of 1.4 MPa, 200 MPa, and 300 MPa: X_c_—crystallinity determined by WAXS, L_p_—average long period determined by SAXS, L_cx_—average lamella thickness based on X_c_ and L_p_, L_av_—average lamella thickness, calculated based on eqs. (3), (5), and (6) according to [48]. (I) denotes samples crystallized isothermally, asterisks mark values based on FS-DSC thermograms. Figure S1a: SAXS patterns of iPP, PP/CN1, PP/CN3, PP/CN5, and PP/MT5 crystallized nonisothermally under 1.4 MPa, 200 MPa, and 300 MPa. Figure S1b. SAXS patterns of iPP and PP/CN5 crystallized isothermally, as denoted by (i), under 200 MPa and 300 MPa. Figure S2: FS-DSC heating thermograms of iPP and PP/CN5 crystallized nonisothermally under 1.4 MPa, 200 MPa, and 300 MPa. Heating rate of 5000 °C/min. Figure S3: True stress–true strain dependencies of PP/CN1, PP/CN3, and PP/MT5 crystallized nonisothermally under 1.4 MPa, 200 MPa, and 300 MPa. Figure S4: SEM micrographs of iPP and PP/CN5 crystallized nonisothermally under 200 MPa and 300 MPa, and compressed to true strain of 0.4: iPP, 200 MPa (a), iPP, 300 MPa (b), PP/CN5, 200 MPa (c), and PP/CN5, 300 MPa (d). LD vertical.
