# Supplementary material for: Structure and Mechanical Properties of iPP-Based Nanocomposites Crystallized under High Pressure
Source: Nanomaterials (Basel). 2024 Apr 4;14(7):629. doi: 10.3390/nano14070629 (PMC11013707; doi:10.3390/nano14070629)
Supplement: Supplementary file 1 [file nanomaterials-14-00629-s001.zip › nanomaterials-2929625-supplementary.pdf]

Supplementary Materials

Structure and Mechanical Properties of iPP-Based Nanocomposites Crystallized under High Pressure

Sivanjineyulu Veluri, Przemyslaw Sowinski, Mariia Svyntkivska, Zbigniew Bartczak, Tomasz Makowski and Ewa Piorkowska *

Centre of Molecular and Macromolecular Studies, Polish Academy of Sciences, Sienkiewicza 112, 90 363 Lodz
* Correspondence: ewa.piorkowska@cbmm.lodz.pl; Tel.: +48-42-6803-316

Table S1. Characteristics of iPP and its nanocomposites, PP/CN and PP/MT5, crystallized under pressure of 1.4 MPa, 200 MPa, and 300 MPa: X_c – crystallinity determined by WAXS, L_p – average long period determined by SAXS, L_{cx} – average lamella thickness based on X_c and L_p, L_{av} – average lamella thickness, calculated based on eqs. (3),(5) and (6), according to [48]. (i) denotes materials crystallized isothermally, asterisks mark values based on FS-DSC thermograms.

Sample code	Pressure (MPa)	X_c (%)	L_p (nm)	L_{cx} (nm)	L_{av} (nm)
iPP	1.4	57	18.2	10.0	10.3, 9.3*
PP/CN1		61	19.4	11.4	10.1
PP/CN3		60	-	-	10.5
PP/CN5		59	-	-	9.7
PP/MT5		57	18.2	10.0	9.3
iPP	200	61	12.9	7.6	8.1, 7.5*
PP/CN1		61	13.9	8.2	8.4
PP/CN3		62	-	-	8.3
PP/CN5		63	-	-	8.3, 8.6*
PP/MT5		53	11.6	5.9	8.2
iPP	300	62	12.3	7.4	8.1, 7.0*
PP/CN1		62	12.7	7.6	7.9
PP/CN3		63	-	-	8.2
PP/CN5		65	-	-	7.9, 6.6*
PP/MT5		57	11.5	6.3	7.9
iPP(i)	200	70	16.9	11.5	10.2
PP/CN5(i)		71	-	-	10.6
iPP(i)	300	69	16.0	10.7	9.7
PP/CN5(i)		71	-	-	9.9

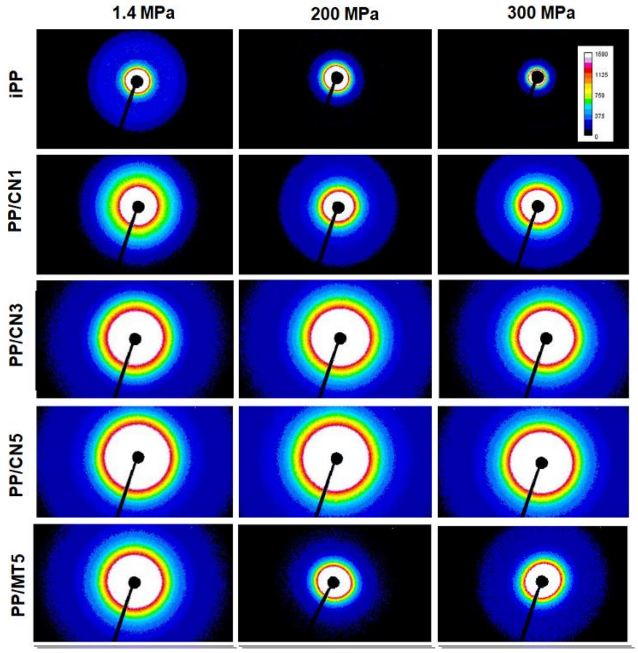

Figure S1a. SAXS patterns of iPP, PP/CN1, PP/CN3, PP/CN5 and PP/MT5 crystallized nonisothermally under 1.4 MPa, 200 MPa, and 300 MPa.

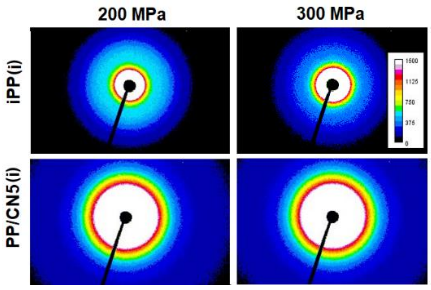

Figure S1b. SAXS patterns of iPP and PP/CN5 crystallized isothermally, as denoted by (i), under 200 MPa and 300 MPa.

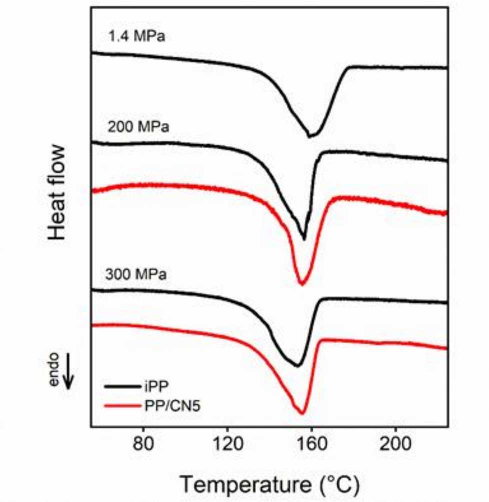

Figure S2. FS-DSC heating thermograms of iPP and PP/CN5 crystallized nonisothermally under 1.4 MPa, 200 MPa and 300 MPa. Heating rate of 5000 °C/min.

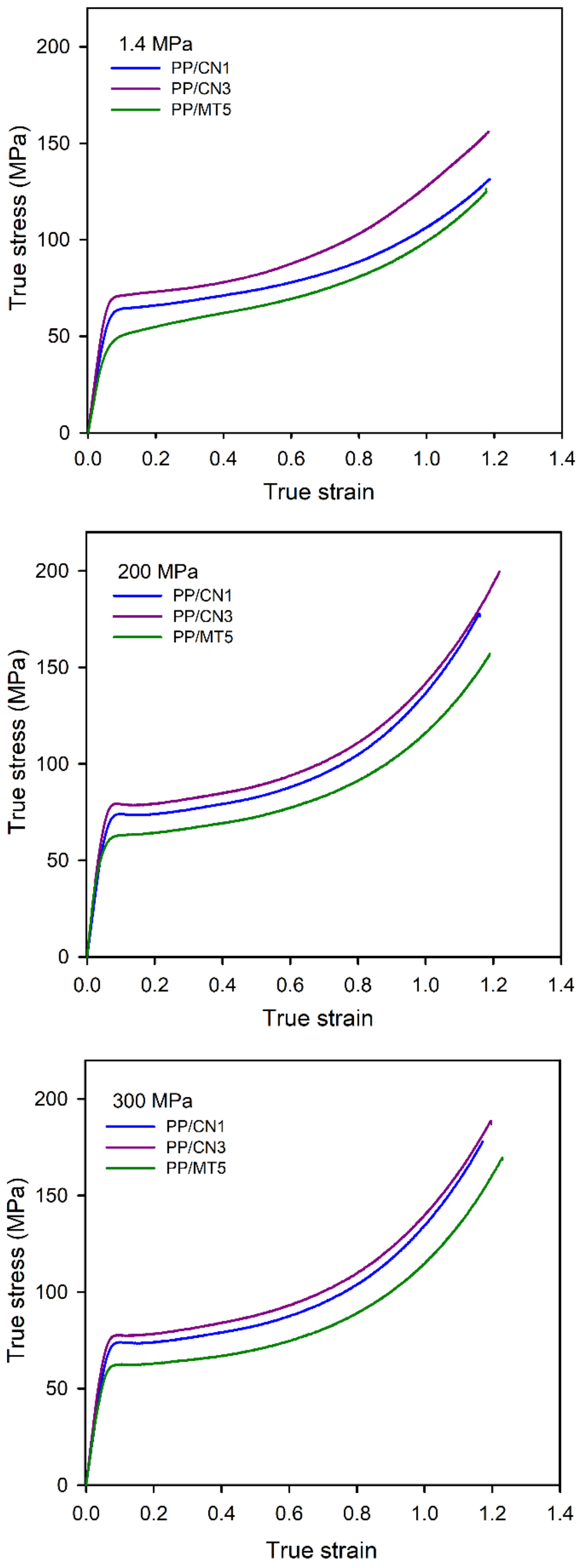

Figure S3. True stress-true strain dependencies of PP/CN1, PP/CN3 and PP/MT5 crystallized nonisothermally under 1.4 MPa, 200 MPa, and 300 MPa.

.

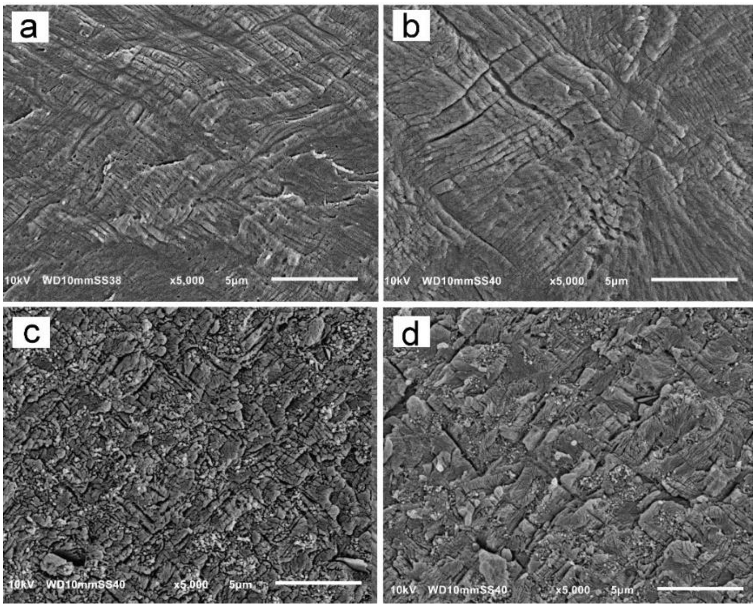

Figure S4. SEM micrographs of iPP and PP/CN5 crystallized nonisothermally under 200 MPa and 300 MPa, and compressed to true strain of 0.4: iPP, 200 MPa (a), iPP, 300 MPa (b), PP/CN5, 200 MPa (c), PP/CN5, 300 MPa (d). LD vertical.
